# Routine Susceptibility Testing of *Helicobacter pylori* in Clinical Practice—Results of a Prospective Multicentre Study

**DOI:** 10.3390/antibiotics15050426

**Published:** 2026-04-23

**Authors:** Anke Hildebrandt, Farina Wewers, Lutz Uflacker, Barbara C. Kahl, Annika Hoyer, Reinhard Bornemann, Markus Brückner

**Affiliations:** 1 Institute of Medical Microbiology, University Hospital Münster, 48149 Münster, Germany; f.wewers@vincenz-datteln.de (F.W.); barbara.kahl@ukmuenster.de (B.C.K.); 2 Department of Internal Medicine I, St. Vincenz-Hospital Datteln, 45711 Datteln, Germany; l.uflacker@vincenz-datteln.de; 3 Biostatistics and Medical Biometry, Medical School OWL, Bielefeld University, 33615 Bielefeld, Germany; annika.hoyer@uni-bielefeld.de; 4 Department of Internal Medicine, Campus Klinikum Bielefeld, University Medical Center OWL, 33604 Bielefeld, Germany; bornemann@uni-bielefeld.de; 5 Department of Population Medicine and Health Services Research (AG2), School of Public Health, Bielefeld University, 33615 Bielefeld, Germany; 6 Department of Gastroenterology, Campus Klinikum Bielefeld, University Medical Center OWL, 33604 Bielefeld, Germany; markus.brueckner@klinikumbielefeld.de

**Keywords:** *Helicobacter pylori*, susceptibility testing, antibiotic therapy, resistance-guided therapy, antimicrobial stewardship, reuse urease biopsies, Germany

## Abstract

**Background/Objectives**: *Helicobacter pylori* (HP) antibiotic eradication treatment in Germany is, at present, empirical according to the national guidelines. The aim of our prospective multicentre study was to implement routine susceptibility testing in daily clinical practice to facilitate resistance-oriented first-line antibiotic therapy and to collect local resistance data. **Methods**: From 1 January 2024 to 30 April 2025, in two German hospitals (in Bielefeld and Datteln), the patients who underwent gastroscopy and those who had biopsies for histopathology also underwent biopsies for the *Helicobacter* urease test (HUT). Positive HUT samples were sent for susceptibility testing: they were checked for phenotypic/cultural resistance to amoxicillin, clarithromycin, metronidazole, levofloxacin, rifampicin and tetracycline and genotypic/molecular resistance to clarithromycin and fluoroquinolones. **Results**: In total, in 1503 cases, both HUT and histology were performed, in which 256 (17.0%) histologies were HP-positive. We sent 311/1503 (20.7%) positive HUTs for susceptibility testing. In 120 (38.6%) of them, it was possible to culture HP, and for 118 cases, phenotypic resistance testing was performed. In 200/311 cases, PCR was also executed, with 111/200 cases being subjected to subsequent molecular resistance testing. Resistance patterns varied regionally, with metronidazole resistance observed in 3–33%, clarithromycin resistance in 16–20% and levofloxacin resistance in 13–16% cases. **Conclusions**: it is technically and logically feasible to perform HP antibiotic susceptibility testing via the same biopsy used for the HUT. The proposed procedures might be applied both in hospital and outpatient settings in endoscopic offices. Routine susceptibility testing is useful not only for the individual patient but also for monitoring the development of regional resistance patterns as a basis for better-targeted empiric therapy. Additionally, this approach might help to reduce the resistance dynamics at large in terms of antimicrobial stewardship.

## 1. Introduction

Infection with *Helicobacter pylori* (*H. pylori*, HP) may cause various gastrointestinal symptoms and diseases, such as B-gastritis, gastric and duodenal ulcers, and gastric neoplasia. Notably, HP infection increases the risk of gastric cancer by 4–6-fold [1], and approximately 80% of all gastric cancers worldwide are estimated to be HP-associated [2].

In Germany, the HP prevalence is estimated to be 35.3% (95-CI 31.2–39.4) [3,4]; however, regional prevalence data are sparse. An ongoing national multicentre study for 2022–2025 found an HP seroprevalence of 19.1% and an HP prevalence of 7.8% in breath tests [5].

HP can be diagnosed using a variety of methods: gastroscopy with biopsies and histopathology—at both study sites this is considered as the reference standard—gastric biopsies for the *Helicobacter* urease test (HUT) and HP culture, polymerase chain reaction (PCR) both from gastric biopsies or stool samples, serology, and the 13 C-breath test. If diagnosed as HP-positive, patients are usually treated with antibiotics (“eradication therapy”). This therapy is based on various combinations of antibiotic substances, including amoxicillin (AMX), clarithromycin (CLR), fluoroquinolones (FQs, especially levofloxacin (LVX)), metronidazole (MTZ), rifampicin/rifabutin (RIF), and tetracycline (TET), together with a proton pump inhibitor (PPI) [3,4].

In Germany, until 2022, the first-line HP eradication scheme was an empirical CLR-based “triple therapy”: either CLR + AMX + proton pump inhibitor (PPI) (“French”) or CLR + MTZ + PPI (“Italian”) [6]. Meanwhile, worldwide, CLR resistance is on the rise, and between 2017 and 2024, CLR-resistant HP was even identified as a pathogen of high relevance [7,8]. A European study reported a CLR resistance rate of 21.4% for Germany during 2008–2017—but only 85 German patients were included [9]. A German study with *n* = 1171 patients reported a CLR resistance rate of 14.5% for 2015–2018 [10]. As an international consensus, the resistance rates of the respective antibiotics for empirical therapy of up to a level of 15% are acceptable in a combined regimen [11].

Consequentially, the national guideline now recommends an empirical treatment with a “quadruple” regimen containing MTZ + TET + PPI + bismuth [3,4]. The reason for this was mainly derived from the results of a European multicentre study in patients with HP infection; of whom 90% were successfully treated with this quadruple therapy. However, it is difficult to extrapolate these results to the local German population, keeping in mind that only *n* = 35 of the total 2100 patients were from Germany [12]. Interestingly, overall, in Europe, even higher resistance rates of 38.9% are known for MTZ—without any specification for Germany [9]. The German data on MTZ resistance are from 2014 [13], whereas more recent data regarding Germany at large are lacking, let alone regional data. However, the resistance rates for CLR and MTZ—and to a lesser extent for FQ/LVX—are of therapeutic relevance, and those for AMX, RIF, and TET are still insignificant [5,9,11,14].

Inadequate use of antibiotics is a key driver of antimicrobial resistance [15], which is becoming an increasingly relevant issue for health in general [15,16] and for HP in particular [6,14,17,18]. Therefore, antibiotic therapies should preferably be targeted and resistance-oriented [18,19,20,21,22]. To cope with inadequate antibiotic use and, in turn, to promote adequate use, a variety of measures were implemented in the past 20 years, under the term “antimicrobial stewardship” (AMS). In Westphalia–Lippe, the north-eastern part of the federal state of North Rhine–Westphalia, many AMS initiatives and efforts were implemented since, e.g., the project “Antibiotic Therapy in Bielefeld” [23,24]. In 2022, some of the Westphalian initiatives formed the “AMS Network Westphalia–Lippe” [25].

HP diagnostics and therapy became a key focus within the AMS network. While the above-mentioned diagnostic tests prove the presence of HP, culture and PCR testing can also reveal the resistance pattern of the respective HP clone. Via culture, susceptibility testing is possible for AMX, CLR, LVX, MTZ, RIF and TET, while genotypic susceptibility testing via PCR can detect resistances for CLR and FQ (including LVX). As an HP infection may undergo antibiotic susceptibility testing, it should be subjected to resistance-guided treatment, as is the case for bacterial diseases in general. Recent scholarship has explored a variety of possible approaches [26], of which a promising selection should now be put into practice in everyday patient care.

With this background of HP resistance, and the pursuit of AMS, the primary aim of our study was to implement a procedure for determining HP resistance in patients with HP infection that was suitable to include in daily routine care, in order to provide the most effective antibiotic treatment for individual patients, to prevent them from experiencing the unnecessary side effects of inadequate antibiotics and to understand resistance dynamics in individuals and in the general population.

Our secondary aims were: (i) to collect HP resistance data at various places and to possibly detect regional variances in resistance patterns as a basis for regionally adapted empiric HP treatment; (ii) to encourage broader HP resistance testing nationwide for epidemiological reasons as a basis for the continuous adaption of national HP recommendations; (iii) to explore combinations of the available HP test methods—HUT, histopathology, culture and PCR—in order to develop HP resistance testing procedures that could be customised to local care conditions.

This led us to ask the following research questions:

- Can commercially available HUTs—beyond an HP detection method—also be used as an intermediate step in re-using the HUT’s bioptic material for an HP culture and/or PCR?
- To what extent do the HP resistance profiles identified in individual patients in Westphalia–Lippe meet the empiric quadruple therapy proposed by the German guideline?
- How is HP resistance distributed in the region of Westphalia–Lippe?

Notably, in this context, HUT was not considered to be a decisive test method for establishing HP infection, but a secondary test to accompany histopathology—as a reference standard—and an intermediate step for further susceptibility testing by culture and/or PCR.

## 2. Results

### 2.1. Case Numbers and Demographics

Overall, *n* = 1503 cases could be included in the analysis, *n* = 355 (23.6%) from Bielefeld and *n* = 1148 (76.4%) from Datteln (Table 1). In total, there were *n* = 705 (46.9%) males and *n* = 798 (53.1%) females, with *n* = 172 (48.5%) males and *n* = 183 (51.5%) females in Bielefeld and *n* = 533 (46.4%) males and *n* = 615 (53.6%) females in Datteln. The mean age of the study participants was 64.3 years (SD: 17.3); in Bielefeld it was 59.0 (18.9) and in Datteln it was 66.0 (16.5). Overall, males were slightly younger compared to females (63.7 [16.6] vs. 64.9 [18.0]).

### 2.2. Basic Population

In both study centres, during the study period, *n* = 7095 OeGDs (OPS 1-632.0) were executed.

### 2.3. Duration of the Various Test Methods

#### 2.3.1. HUT Duration

The duration of both test kits (the one used in Bielefeld, with a duration of up to 60 min, and the one used in Datteln, with a duration of up to 15 min) was short and did not differ substantially.

#### 2.3.2. Histology Duration

In *n* = 572 cases for which a histology duration was available, the majority of the results (*n* = 256) were available within one day, *n* = 116 within two days, *n* = 96 within three days, and the remaining within up to 18 days (including weekend days and longer-lasting durations, e.g., due to more complex histologies; with no relevant differences between both study sites).

#### 2.3.3. Culture Duration

The culture duration was more widespread, ranging from a minimum of six days (in one case) to a maximum of three weeks or more (in four cases), with a median duration of 12.0 days (Q1, Q3 10.0/14.0). In Bielefeld, in 9/10 cases, the specimen designed for culture reached the laboratory on the day of the gastroscopy. In the remaining cases it usually reached the laboratory on the next workday, whereas in Datteln, samples were sent via the hospital’s reference laboratory in Münster to another laboratory in Heidelberg, which took 3–5 days. According to the respective laboratories, this variation also depended on the bacterial load within the specimen—the higher the load, the shorter the duration, and vice versa.

#### 2.3.4. PCR Duration

The PCR duration, which was only applied in Datteln, cannot be described separately, as culture and PCR test results were received in a combined laboratory report.

### 2.4. Test Results According to the HP Test Method

Following the HP records according to the various test methods—HUT, histology, culture and PCR—were described (Table 2).

#### 2.4.1. Histology (Reference Standard)

Histology was performed in all 1503 cases: HP-positive in *n* = 256 (17.0%) and HP-negative in *n* = 1247 (83.0%) cases.

#### 2.4.2. HUT (Index Test)

HUT was performed in all 1503 cases: HP-positive in *n* = 322 (21.4%) and HP-negative in *n* = 1181 (78.6%) cases.

#### 2.4.3. Culture (Index Test)

Culture was performed in 311 cases: HP-positive in *n* = 120 (38.6%) and HP-negative in *n* = 191 (61.4%) cases.

#### 2.4.4. PCR (Index Test)

PCR was performed in 200 cases: HP-positive in *n* = 111 (55.5%) and HP-negative in *n* = 89 (44.5%) cases.

#### 2.4.5. Comparison of HUT vs. Histology

In comparison to the reference standard HP-histology, the raw sensitivity and specificity of the HUT were 71.9% (95–confidence interval: [66.1; 77.00]) and 88.9% [87.1; 90.6], respectively. The GLMM, accounting for potential correlations between the different index tests and the study centres, revealed a sensitivity of 74.0% [66.8; 81.2] and a specificity of 90.9% [87.7; 94.0]. Adjusting for age and sex, an overall sensitivity of 76.3% [65.9; 86.7] and a specificity of 92.0% [87.4; 96.6] was found.

#### 2.4.6. Comparison of Culture vs. Histology

Contingency tables led to a raw sensitivity of 64.9% [57.5; 71.7] and specificity of 93.6% [88.1; 96.6] for culture. Based on the GLMM, we estimated a sensitivity of 71.7% [63.5; 80.0] and a specificity of 89.9% [85.3; 94.5]. The findings from the sensitivity analysis were slightly higher, but comparable: 74.2% [62.8; 85.5] for sensitivity and 91.1% [85.4; 96.9] for specificity.

#### 2.4.7. Comparison of PCR vs. Histology

The raw sensitivity and specificity of the PCR were 94.9% [88.3; 97.9] and 82.4% [73.7; 88.6]. The GLMM led to a sensitivity of 86.3% [79.7; 92.9] and a specificity of 95.7% [93.0; 98.4]. Adjusting for age and sex in a sensitivity analysis resulted in a sensitivity of 87.7 [80.1; 95.3] and a specificity of 96.2% [93.3; 99.2].

### 2.5. Resistance Patterns

#### 2.5.1. Cultural Resistance

In *n* = 118 HP-positive cultures (Bielefeld *n* = 60, Datteln *n* = 58), resistance patterns could be acquired (in *n* = 2, this was technically not possible). For cultural resistance patterns, see Table 3.

For culture, based on a logistic regression model that also adjusts for age and sex, we could confirm that the odds for CLR resistance are lower for Datteln than for Bielefeld (odds ratio [OR] = 0.42, 95-confidence interval = [0.21; 0.65]). The same is true for LVX (OR = 0.53 [0.29; 0.77]) and MTZ (OR = 0.06 [0.01; 0.21]).

#### 2.5.2. PCR Resistance

In total, *n* = 111/200 PCR tests were HP-positive and also yielded results for CLR and FQ resistance. In *n* = 82 cases, there was no resistance for both CLR and FLQ. A single instance of CLR resistance was found in *n* = 11 cases, and a single instance of FQ resistance was found in another *n* = 11 cases. Double CLR and FQ resistance was found in *n* = 5 cases. In *n* = 2 cases, one with and one without CLR resistance, FQ resistance could not be determined (Table 4).

For the PCR results, a logistic regression analysis that adjusts for age and sex indicated lower odds for CLR resistance for women compared to men (OR = 0.78 [0.54; 0.93]). A comparable result was found for FQ resistance (OR = 0.56 [0.30; 0.79]). Additionally, our data indicated that the odds of CLR or FQ resistance decreased with increasing age (OR = 0.50 [0.49; 0.51] and OR = 0.50 [0.49; 0.51], respectively).

#### 2.5.3. Cultural vs. PCR Resistance

In *n* = 89/200 cases with PCR, concordantly, culture and PCR were HP-negative. In the remaining *n* = 111 that were PCR-positive with resistance information, the concordance and, respectively, discordance with culture was as follows: In *n* = 53 cases, the discordantly culture was negative vs. PCR-positive; thus, no resistance concordance or discordance could be determined. In *n* = 42 cases, concordantly, in both culture and PCR, HP was sensitive both to CLR and FQ/LVX. In *n* = 5 cases, concordantly, HP was resistant to CLR in both culture and PCR, and in another *n* = 5 cases it was concordant with FQ/LVX. In *n* = 3 cases, CLR and FQ/LVX were concordantly resistant in both culture and PCR. Discordantly, in culture, in one case, CLR and LVX were resistant vs. sensitive in PCR, and in culture, in one case, LVX was sensitive vs. FQ-resistant in PCR (Table 5).

Regarding the question of whether study patients were treatment-naive or had received prior HP eradication therapy, in Bielefeld, this history was not recorded systematically. In Datteln, in 14 of 200 (7.0%) patients, a previous HP eradication therapy was reported, and in 10 of these the respective antibiotic regimen was identified. In 5 of these 10, both culture and PCR were HP-negative. Of the remaining five patients who were HP-positive, two showed resistances (1x LVX, 1x RIF). Thus, 106 of the available 111 HP susceptibility patterns in Datteln were attributed to therapy-naive patients.

#### 2.5.4. Empiric Quadruple vs. Resistance-Guided Therapy Regimens

We compared the appropriateness of an empiric quadruple therapy with the therapeutic options of the resistance profiles obtained in individual patients. In Bielefeld, in *n* = 20/60 (33.3%) HP-positive cultures with mono, double or triple resistances towards MTZ, the empirical MTZ-containing quadruple scheme did not seem appropriate. Otherwise, the CLR-based triple schemes previously recommended by the guidelines in only *n* = 12/60 (20.0%) cases with CLR resistances were not appropriate according to the individual resistance patterns. In Datteln, *n* = 3/58 (5.2%) HP-positive cultures showed resistance against MTZ, and thus were not suitable for quadruple therapy, whereas *n* = 12/58 (20.7%) showed CLR resistance precluding CLR-based regimens. In the *n* = 111/200 HP-positive PCRs, which underwent genotypic resistance testing against CLR and FQ, *n* = 17/111 (15.3%) CLR-resistances precluded CLR-regimens.

## 3. Discussion

### 3.1. Methodological Considerations

One of the initial challenges we faced in our study was implementing a new procedure in the endoscopic departments of both study sites. First, the staff had to be instructed, both personally and by providing information material. Second, the required test material—HUT and culture medium—had to be identified and obtained. Third, the workflow in daily practice had to be elaborated upon, including the documentation of the procedure and shipping of the culture medium. Finally, attention had to be given to the return flow of the results and their transfer into individual patient care. In principle, all of this had already been achieved during the pilot studies, when both study sites were in close exchange. However, during the study period, some possible pitfalls emerged that had to be addressed. Overall, though, the procedure ran smoothly at both study sites.

The marked inter-centre difference in culture positivity rates (approx. Bielefeld 59% vs. Datteln 29%) must be questioned. First, the patients’ characteristics may have differed, as in Bielefeld the HUT application was selective with regard to gastroscopic findings, with a higher probability of HP infection. Second, and perhaps most importantly, in Bielefeld, culture samples usually reached the microbiological laboratory on the same day, whereas in Datteln this took 3–5 days, which had a negative effect on culturability. The fact that the culture positivity rate of 59.0% in Bielefeld was comparable to the PCR positivity rate in Datteln at 55.5%—noting that PCR seems to be more “time-robust” (though PCR was not performed in Bielefeld to allow a direct comparison)—suggests that the long transfer time in Datteln may have played an unfavourable role. Finally, the analytical handling at both microbiological laboratories was comparable, with only slight differences. However, culturing in Datteln was discontinued after 7 days, whereas in Bielefeld it was stopped after 14 days. This may also have contributed to the lower cultural detection rate in Datteln.

For this reason, we state that in each setting—outpatient or hospital care—such logistic conditions are relevant to the decision to perform HP resistance testing either via culture or by PCR. In addition, the genotypic testing by PCR assays used in the study only detected mutations associated with CLR and FQ resistance. Genotypic/PCR assays covering the relevant MTZ resistance have already been applied [27,28]; however, they do not seem to be broadly available. In contrast, phenotypic testing covers resistances to all six drugs that can be used for treatment. Thus, PCR provides only a partial picture of resistance patterns and therefore complements rather than replaces culture-based susceptibility testing. In addition, genotypic resistance to MTZ is complex, involving a multitude of genes with various predictivity on phenotypic MTZ resistance [11].

It should also be noted that the cultural resistance rates for MTZ differed markedly between the two study centres, with the respective differences for CLR and FQ being much smaller. The reason for this discrepancy remains unclear and might reflect different patient populations, different local antimicrobial treatment strategies or methodological aspects of MTZ susceptibility testing, despite standardised procedures. Differences between phenotypic and genotypic resistance results could be due to several underlying factors such as heteroresistance of strains, silent mutations that do not lead to resistance despite the presence of the resistance gene, or mixed infections.

The possibility of reusing gastroscopic biopsies after HUT for susceptibility testing has already been applied for PCR [10,29], whereas we could demonstrate that this also works for culture, thus broadening the options that are locally available. However, only a few laboratories offer culture and/or phenotypic sensitivity testing of HP. Some of the reasons for this include the high material and personnel costs, the need for special grow media, and the non-automatised susceptibility tests. Therefore, from Datteln, we had to send our biopsies via Münster to Heidelberg. In Germany, microbiological HP diagnostics are strongly underfinanced [26]. Thus, routine susceptibility testing requires established and well-reimbursed access to culture or PCR for both outpatient and hospital medicine.

Future works should evaluate whether separate biopsies for susceptibility testing should be processed by HUTs first and then be translocated to transport medium following a positive HUT result or whether they should be primarily located in medium and then stored until the retrieval of a positive histology result. The advantage of the former is the availability of an independent HP result and less handling. The advantage of the latter is the elimination of extra-handling in the individual, for the price of extra-handling in general, besides the logistic/storage and resource/environmental considerations.

A relevant limitation is the non-systematic patient selection, especially in Bielefeld, where HUT and resistance testing were preferably performed in patients with abnormal macroscopic findings. This proceeding implicates potential biases, including enhanced pretest probability of an HP infection. However, it was not the intention of the study to acquire HP prevalence data, but to attain experience with HP resistance testing approaches for their possible implementation in everyday clinical practice. In this context, it also has to be discussed whether the HP resistance prevalence estimates could be regarded as representative. It might be assumed that the yield of HP detection—and consecutively that of cultural resistance findings—would have been considerably lower in normal gastroscopic sights and thus might not have distorted the true resistance prevalence in a relevant way. In addition, the latter aspect does not affect the individual treatment choice based on an individual’s resistogram. It, however, becomes relevant when selected resistance data should be used for empirical treatment recommendations in such patients in whom no resistance testing is available. Therefore, it would be desirable to expand resistance testing in order to get more representative resistance data as a basis for empirical treatment recommendations.

### 3.2. Validity of Test Results

As the use of HUT results was a core element of this study, the validity of HUT results—vs. the reference standard histopathology—should be discussed.

False-negative HUT results were found in *n* = 72/256 (28.1%) cases. There are various reasons why false-negative results may occur: the lower sensitivity of POCT in general and the susceptibility of the HUT procedure to the individual skills in particular, with the performance of endoscopic staff being one example. Yet false-negative HUTs do not lead to worse treatment, as at least histology should become positive, and in consequence the patient will receive empiric eradication treatment—which, however, might be adapted to local resistance patterns.

False-positive HUT results we found in 138/1247 (11.1%) cases. This might be due to the fact, that histology, as the reference standard, could be false-negative, for example in such cases, where, by chance, the biopsies going to histology were taken from gastric areas with a lower HP density and the biopsies for HUT were taken from areas with a higher HP density. A rare scenario might be the insufficient processing of the biopsies in the pathology department. Also, the positive reading of the HUT result might have been too sensitive when, in fact, it should have been read as negative, e.g., in rather bloody biopsies. When all three HP test methods—histology, culture and PCR—are performed at the same time, and one or more out of them is positive, then a false-positive HUT is rare [30], but this scenario is less realistic. The clinical consequence of false-positive HUTs seems not to be relevant in most cases, as a negative histology result will neutralise a contrasting HUT result. If, based on a positive HUT, susceptibility testing is performed (culture or PCR), the respective results will enlarge the picture. In HUT-positive cases where HP results of histology and susceptibility tests are contrary, we suggest starting HP treatment; preferably resistance-guided treatment, if available, and otherwise empirically.

The relevance of the underlying gastric entity, e.g., gastritis, gastric or duodenal ulcer, etc., for either false-positive or false-negative HUT results will be evaluated in a follow-up study.

Taken together, first, the HUT results show an agreeable accordance with the histology standard, and second, there was also an accordance between histology, culture and PCR. The discordant cases with each negative histology or culture could be attributed to different bacterial densities at the biopsy sites, and—facing the preponderance of HP-positive histologies—technical issues in the culture pre-analytics. Here, the observed transport delays of several days in Datteln vs. in Bielefeld are incompatible with optimal *H. pylori* viability, and might have led to lower cultural detectability, as well as the shorter incubation time of 7 days in Datteln vs. 14 days in Bielefeld. However, this study was initiated to show the possible options for available resistance testing in practical work, not to compare the validity of various test methods.

It is relevant to mention here that non-*Helicobacter pylori Helicobacter* species (NHPH) have received increasing attention in recent years [31]. Although the NHPH prevalence seems low, the potential impact of such species on HP diagnostics and resistance patterns should be considered in the future.

There have been discordances between phenotypic (culture) and genotypic (PCR) results and resistance patterns (“internal validity”) in three cases: #1 phenotypic resistance vs. genotypic sensitivity towards CLR; #2 phenotypic resistance vs. genotypic sensitivity towards FQ/LVX; #3 phenotypic sensitivity vs. genotypic resistance towards FQ/LVX.

From the multicentre HelicoPTER study, coordinated by the National Reference Centre *Helicobacter pylori* in Munich, preliminary data from 2025 of 5750 participants show that the resistance towards CLR was 18.3%, to LVX it was 15.9%, and to MTZ it was 42.5%, whereas RIF had 5.8% resistance and AMX and TET each had 0.0% resistance [5]. Further results of this study may provide more regional resistance data for locally adapted empirical treatment.

Data from two big laboratories covering the Bielefeld area in 2019–2024 show almost 100% sensitivity of HP against AMX, RIF and TET, compared to sensitivity of around 20% for LVX, 50% for CLR and 60% for MTZ [32]. These data sets cannot be easily compared with our results, as in the past HP resistance testing was usually performed only after failure of preceding empiric therapies.

Embracing the resistance data from Germany already depicted in the introduction, it can be noted that there is a relevant regional—and to a lesser degree temporal—variation in HP resistance patterns so far.

Notably, our data does not provide sufficient support for the theory that MTZ resistance observed in vitro reliably predicts treatment failure in vivo, and therefore makes empirical bismuth quadruple therapy containing MTZ unsuitable in a considerable proportion of patients. In these quadruple regimens, the impact of MTZ resistance might be at least partially overcome.

### 3.3. Clinical Considerations

Whereas PCR testing might be performed using the histopathological material, HP culture requires two additional gastric biopsies (from the antrum and corpus), which might increase the bleeding risk associated with the procedure. However, in many cases, gastroscopies lead to a significant number of additional biopsies due to suspicious mucosal areas without considering an increased bleeding risk, assuming normal coagulation in the patient. During our study, no bleeding complications were documented. The slightly longer time taken for the additional biopsies, estimated at under one minute, should not pose an additional risk. Therefore, the additional biopsies seem to be justified in order to enable resistant-appropriate treatment.

With the use of the HUT in combination with histology, it is possible to fulfil the two postulated tests to prove the presence of HP infection according to the DGVS [3,4]. Following this, our procedure turned out to be a good tool to preselect patients, so that we only sent biopsies from positive HUTs to the microbiological laboratory. Furthermore, without relevant additional endoscopic time, the daily workflow in the endoscopy was not disturbed. Another working group from South Korea recently published a similar approach in which they achieved successful HP culture and susceptibility testing in approximately 80% of cases using tissue samples obtained both before and after HUTs [33].

Further, we would like to raise some questions about the test strategy for future routine practice. What criteria should be used to determine whether biopsies should be sent for susceptibility testing: HUT positive (as in our study), clinical and endoscopic criteria (e.g., anamnesis, ulcera) or a combination of criteria? Would it be practical to reuse biopsies after HUT testing? The use of two different commercially available HUTs in routine application during gastroscopy proved to be a feasible approach.

In cases where biopsies were sent for susceptibility testing, medical practitioners needed to be informed to wait for the resistance patterns to emerge before starting an empiric eradication therapy, as before. We informed all practitioners at both study centres that biopsies had been sent for further resistance analyses and that detailed recommendations for resistance-guided therapy would be provided at a later date. In a noteworthy proportion of our patients, the currently recommended empirical first-line “quadruple” therapy would not have been suitable for susceptibility test results. A follow-up study is scheduled and will comprise resistance profiles, therapy recommendations by us, consecutive prescriptions by the GPs and the eradication outcome in individual patients, as well as the structural implications of this sequence chain.

In cases where no resistance information is available, as was the case with the different resistance patterns detected at both study sites, regional empirical recommendations based on regional HP resistance data seem to be more desirable than national empiric recommendations.

In our study, we encountered obstacles to routine resistance-guided therapy: (a) HP resistance diagnostics are currently underfinanced in Germany [26]. (b) Logistic structures need to be built up. (c) Diagnosis of HP infection usually takes place in hospitals or gastroenterological practice, e.g., in two different sectors of the German health system with different organisation and financing.

With a positive HUT, a prognosis becomes available which suggests that the respective patient has an HP infection and thus might be eligible for HP eradication therapy after this HP-positive status has been confirmed by the reference standard histopathology. With the demonstrated prediction probability of the HUT result, the same biopsy material that has already been used for the HUT should be transferred into an appropriate medium, such as PortPyl^®^, for susceptibility testing.

The respective bioptic material, if properly treated, yields good results in further susceptibility testing either by culture or by PCR. However, continuing routine practice of performing the HUT and transferring it via PortPyl^®^ to the laboratory showed that this procedure is subject to preanalytical precautions; that is, in a number of cases, the culture was not successful as mentioned above (whereas PCR still might be performed).

A delay of around two weeks until the receipt of an HP resistogram after culture seems to be tolerable by clinical considerations, as an HP infection is a chronic condition, and thus resistance-guided antibiotic therapy should be given preference. In the case of susceptibility testing by PCR, the delay is only up to one week. Indeed, patients usually receive PPI treatment immediately after the detection of gastric pathology in order to relieve their symptoms. However, it should be taken into account that this delay requires some extra logistics by transmitting the resistance testing results to the GP who—in the German context—prescribes the eradication treatment. Whereas the information of a detected HP infection in the discharge letter with a recommendation for an empiric HP treatment is simple, the delayed transmission of the respective resistance information, possibly leading to a divergent treatment, is somewhat challenging.

Meanwhile, we started a follow-up study in order to obtain information about the GPs’ respective eradication prescriptions and the prevalence and results of the control of eradication efficacy.

## 4. Materials and Methods

### 4.1. Study Type

The study was a prospective multicentre study at two study sites: Klinikum Bielefeld, Germany, and St. Vincenz-Hospital Datteln, Germany.

### 4.2. Setting, Study Centres

Bielefeld is a large town in the eastern part of the federal state of North Rhine–Westphalia (NRW), with approximately 330,000 inhabitants. The Campus Klinikum Bielefeld is a maximum care hospital with 1100 beds and since 2021 it has been part of the University Hospital OWL. Datteln is a city in the centre of NRW with approximately 35,500 inhabitants. The St. Vincenz-Hospital is a basic and standard care facility of the Vestische Caritas-Kliniken with 330 beds. Since 2023, the hospital has served as an academic teaching hospital of the Medical Faculty of the Ruhr University Bochum.

### 4.3. Study Period

The study period of the main study was from 1 January 2024 until 30 April 2025 (Datteln: 1 January 2024–31 March 2025, Bielefeld: 1 February 2024–30 April 2025).

### 4.4. Inclusion/Exclusion Criteria

In principle, all adult patients aged 18 and over could be included, if they had undergone a diagnostic gastroscopy (respectively, oesophagogastroduodenoscopy, OeGD, OPS 1-632.0) to investigate abdominal symptoms, or for follow-up after previous diagnosis of a gastroduodenal disease. All patients under 18 years of age were excluded.

### 4.5. Laboratory Tests

In order to preselect the gastric biopsies with high HP probability, we used HUT as an indicator test: when positive it led to culture (at both study sites) and PCR (in Datteln only). In all cases, histology was carried out as the reference standard.

#### 4.5.1. HUT

At both study sites, commercially available *Helicobacter* urease rapid tests (HUTs) were used: in Bielefeld, the test used was Dry^®^ (MIC, Herford, Germany), and in Datteln, the *Helicobacter* pylori quicktest (KeySurgical^®^, Lensahn, Germany) was used.

#### 4.5.2. Histopathology

Gastric biopsies at both study sites were routinely stained with haematoxylin–eosin and Giemsa. In Datteln, the Warthin–Starry silver stain was used as described by Pernick [34]. The histopathological processing of gastric biopsies is to be considered the reference standard, as it can provide information on both the disease entity or aetiology—such as HP colonisation/B-gastritis—and the severity of the disease. However, the immediate placement of the biopsies during gastroscopy into formalin—due to its bactericidal properties—makes additional microbiological examination via culture and resistance testing impossible. However, molecular genetic processing with genotypic resistance testing is in principle possible.

#### 4.5.3. Culture

In vitro viability of HP is difficult to maintain. Therefore, a special transport medium from the endoscopic site to a microbiological laboratory is required (e.g., Portagerm pylori (PORT-PYL^®^), 2.5 mL vial, bioMérieux, Nürtingen, Germany). PortPyl^®^ has a limited stability time (approx. 1 month), so storage and supply have to be monitored regularly.

The subsequent cultivation or molecular genetic processing of the biopsies was carried out in Bielefeld by Labor Krone, Bad Salzuflen, and in Datteln by Labor Dr. Limbach, Heidelberg. Both microbiological laboratories cultured HP according to the German Microbiological Standard “MIQ 09: Gastrointestinale Infektionen” [35].

HP was cultured on commercially available selective media (Oxoid™ Selective Agar, ThermoFisher Scientific, Darmstadt, Germany; it is selective against *Candida* species due to the addition of cefsulodin and amphotericin B). *Helicobacter* agar plates were brought to room temperature before cultivation under laminar air-flow. The biopsies were taken out of the transport medium and put in a separate tube before being homogenised with a mortar. Crushed biopsies were smeared in a grass-like form on agar plates, then the plates were put headfirst in an anaerobic pot with a GasPak™ EZ Campy Container System (Becton Dickinson, Franklin Lakes, NJ, USA) at 35–37 °C. Readings were performed twice weekly, but after 3 days at the earliest. The final reading was performed after 7 days in Datteln and 14 days in Bielefeld, respectively.

In the case of HP growth, in the suspected clones, the following tests were performed: Gram stain (microscopy: curved, Gram-negative rods), catalase (HP: catalase-positive), oxidase (HP: oxidase-positive), species differentiation with MALDI-TOF (in Datteln in all cases; in Bielefeld in rare uncertain cases only).

Susceptibility testing was performed using Epsilometer-(E-)test strips (MIC Strip Liofilchem S.r.l., Roseto degli Abruzzi, Italy) for the six antibiotics that could be used for HP-treatment: AMX, CLR, LVX, MTZ, RIF, and TET. Agar plates were preheated. A bacterial suspension was produced in 2.5 mL 0.9% NaCl-solution and spread on the plates with a cotton swab. The incubation period was 3 days under microaerophilic conditions, e.g., in an anaerobic pot with GasPak™ EZ Campy Container System (Becton Dickinson, Franklin Lakes, NJ, USA) on 35–37 °C. MTZ was incubated for one day anaerobically with Oxoid™ AnaeroGen™ 2.5 L (ThermoFisher Scientific, Darmstadt, Germany) on 35–37 °C and afterwards for two days under microaerophilic conditions (interpretation of the susceptibility test according to EUCAST [36], Table 6).

#### 4.5.4. Molecular Genetics

Molecular diagnostics were performed via PCR to detect genotypic resistance to CLR (23S rRNA gene) and FQ (gyrA gene) (GenoType^®^ HelicoDR test, Bruker, Nehren, Germany).

### 4.6. Pilot Study

A pilot study was conducted at both study sites: (a) to explore diagnostic options of the combinations of HUT and culture or PCR, (b) to establish the respective procedures and logistics for the main study, and (c) to get an initial impression of the local HP resistance situation.

### 4.7. Main Study Procedure

During gastroscopy, and after the decision was made to perform a biopsy for histopathology, each biopsy from the standard sites in the antrum and the corpus was taken for the HUT. Afterwards, the routine biopsies for histology (from the antrum and corpus) were acquired. The HUT biopsies had to be done before the histology biopsies because the latter are dropped into formalin, thus contaminating the biopsy forceps, as formalin is known to disturb further cultivation and genotypic analysis [27]. Both HUT biopsies were transferred directly from the forceps to the HUT. Within the indicated time, according to the manufacturer’s instructions, the HUT was read. If it was HP-positive, a PortPyl^®^ test tube was taken out of the refrigerator (temperature 2–8 °C) and brought to room temperature. Next, the carrier platelet of the HUT was transferred to the PortPyl^®^ test tube and submerged in the medium. Afterwards, the tube was kept at room temperature and sent to the respective microbiological laboratory.

**Figure 1:**
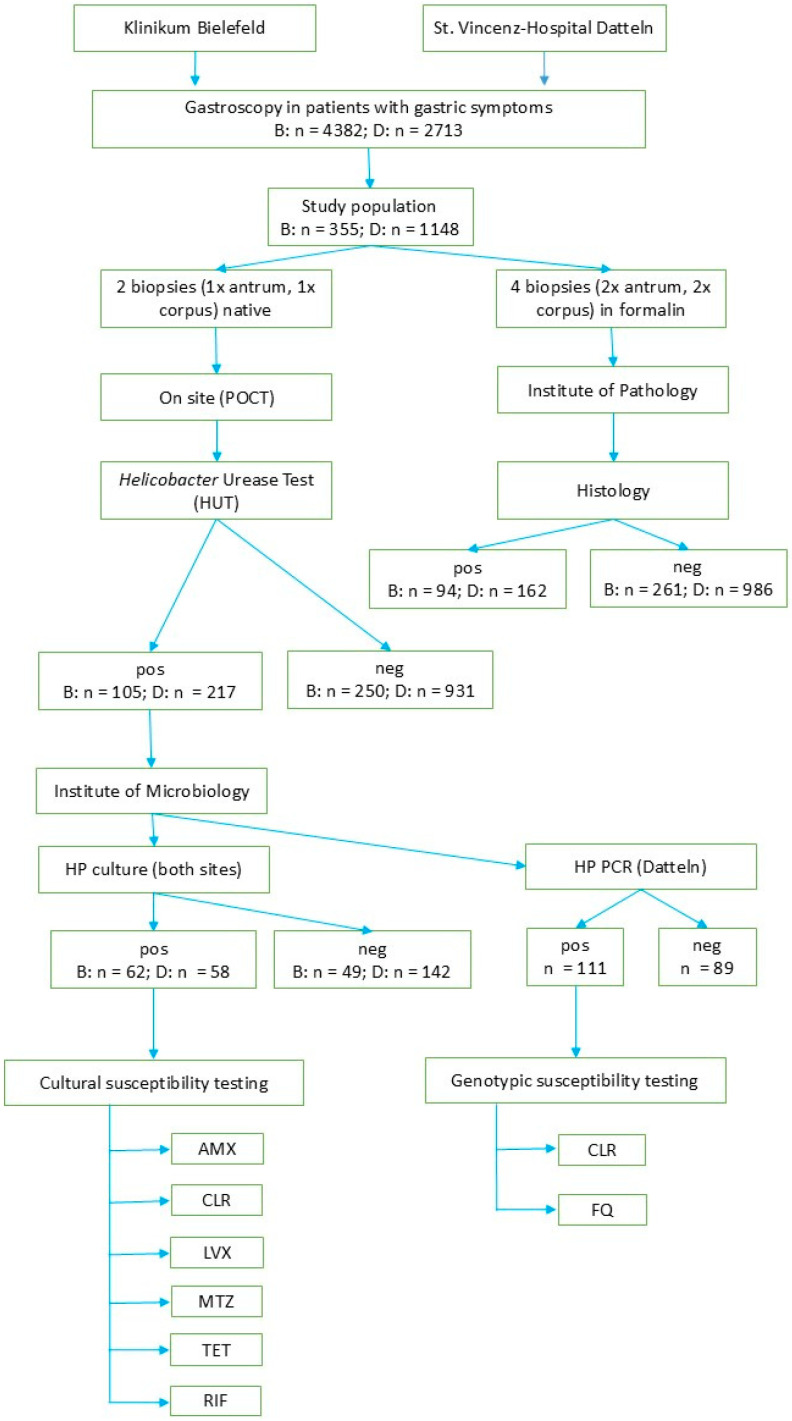
Workflow of HP testing.

In Bielefeld, as a large number of gastroscopies was expected during the study period and financial and personnel resources were limited, a selection of patients had to be taken. A major goal of the study was to explore the feasibility of HP resistance testing in daily practice. As there was a higher pretest probability of HP infection in abnormal macroscopic findings, such as inflammation or ulcer, in these cases, the HUT—and if positive, consecutive culture—was performed preferentially. This increased the yield of positive HUTs and consecutive successful cultures/resistograms. In Datteln, with an overall number of 2713 gastroscopies performed during the study period, in all 1155 cases with biopsies for histopathology, HUTs were also performed. Of these, 217 turned out to be HP-positive. In these cases, principally, subsequent culture and PCR should have been performed; this could have been achieved in *n* = 200/217 (92.2%). In the remaining 17 cases, this was not done, e.g., because of emergency gastroscopies, unstable patients with reduced timeline for gastroscopy, or patients with increased bleeding risk which posed a limitation for biopsies (Figure 1).

### 4.8. Data Collection and Statistics

The primary data was locally collected in MS Excel. The statistical analysis was performed in the statistical software R (version 4.5.2).

As this study is a non-confirmatory pilot study (proof-of-concept study), and there was no preliminary work or other publications on which to base it, it was not possible to carry out any explicit sample size planning beforehand.

The data was first analysed descriptively with respect to the variable type: mean and standard deviation for continuous variables and numbers and proportions for categorical variables. To assess the sensitivity and specificity of the different diagnostic tests (HUT, culture and PCR) in comparison to histopathology (reference standard), we used a logistic regression model with random intercept (GLMM) to account for potential correlations between the three index tests (paired design) and the study centre as a cluster. As the outcome, we modelled the agreement between the different index tests and the reference standard to estimate the sensitivity and specificity for each index test with a 95% confidence interval, and adjusted for the paired study design. In a sensitivity analysis, we also adjusted the model for age and sex. To further describe cultural and PCR resistance patterns, we used logistic regression models with the occurrence of a specific resistance (CLR, LVX, MTZ) as the outcome, and study centre, age and sex as covariates, where appropriate.

We oriented our manuscript according to STARD [37].

## 5. Conclusions

HP antibiotic susceptibility testing, either by culture or PCR, is available in microbiological laboratories. Susceptibility testing offers individual resistance patterns as a basis for individual resistance-tailored antibiotic therapy. In our study, we demonstrated the feasibility of implementing various approaches to HP susceptibility testing in daily clinical practice. According to our results, in a relevant number of patients, no resistances were found, thus allowing the complete spectrum of established eradication regimens, instead of an undirected empiric treatment.

In cases without CLR resistance, conventional CLR-containing regimens can be used. In contrast, if MTZ resistance is present, the appropriateness of an MTZ-containing bismuth quadruple regimen might be discussed. This resistance-oriented treatment approach should increase the therapeutic effects and reduce the side effects of inappropriate antibiotic substances. Nonetheless, this remains to be substantiated by further outcome studies comparing susceptibility-adjusted and empirical based therapy regimens.

In addition, from a public health and AMS perspective, resistance-guided HP eradication therapy might reduce resistance dynamics and thus should also be favoured. Moreover, broader HP resistance testing, preferably in therapy-naive patients, will lead to population-based resistance data and thus might have an impact on further empirical guideline recommendations for those patients in whom susceptibility testing is not available. Altogether, efforts concerning HP sensitivity testing and subsequent sensitivity-adapted treatment should be integrated in a comprehensive AMR/AMS concept within respective institutions, as well as in outpatient care as in the hospital.

### Practical Recommendations

- Endoscopic offices should explore the availability and practicability of the various HP susceptibility testing methods to identify the most suitable method for the local setting.
- At the diagnostic site, sensitisation and education of the staff in the respective testing method, as well as the logistics—especially the storage of test kits and timely transport to the laboratory—should be established.
- During gastroscopy with biopsies, a susceptibility testing rationale is necessary, either comprising all patients or ensuring that they are selected according to pathological findings.
- All players involved should communicate in a way that is continuously evaluated. This applies firstly to the communication between the diagnostic site and the laboratory, and secondly to the communication between the diagnostic site and the subsequent outpatient medical care.
- “Top-down” guidelines should propagate HP susceptibility testing, where available, as the preferred approach to allow resistance-oriented eradication therapy.
- Local or regional physicians’ networks (“bottom-up”) should share the respective information and encourage resistance-oriented HP eradication therapy both in the outpatient and hospital sectors.

## Figures and Tables

**Table 1:** Demographics of the study population.

		Bielefeld		Datteln		Total	
		*n*	%	*n*	%	*n*	%
participants		355	23.6	1148	76.4	1503	100
sex							
male		172	48.5	533	46.4	705	46.9
female		183	51.5	615	53.6	798	53.1
age range (yrs)		18.5–93.3		18.0–100.9		18.0–100.9	
mean		59.0		66.0		64.3	
SD		18.9		16.5		17.3	
age males	mean					63.7	
SD						16.6	
age females	mean					64.9	
SD						18.0	

**Table 2:** Test results according to the HP test methods.

Test Method	Bielefeld		Datteln		Total	
	*n*	%	*n*	%	*n*	%
HUT	355		1148		1503	
neg.	250	70.4	931	81.1	1181	78.6
pos.	105	29.6	217	18.9	322	21.4
histology	355		1148		1503	
neg.	261	73.5	986	85.9	1247	83
pos.	94	26.5	162	14.1	256	17
culture *	111		200		311	
neg.	49	44.1	142	71	191	61.4
pos.	62	55.9	58	29	120	38.6
PCR **					200	
neg.					89	44.5
pos.					111	55.5

* In Bielefeld *n* = 6 and in Datteln *n* = 17 probes went missing or could not be analysed for technical reasons; in Bielefeld *n* = 12 probes were sent for culture testing despite a negative HUT because of the morphological assumption of the HP relation; ** PCR was only performed in Datteln; pos.—positive; neg.—negative.

**Table 3:** Cultural resistance patterns.

	Bielefeld		Datteln		Total	
	*n*	%	*n*	%	*n*	%
HP pos. cultures	60		58		118	
AB resistances						
no resistance	30	50.0	36	62.1	66	55.9
any resistance	30	50.0	22	37.9	52	44.1
single resistance						
AMX	0	0	0	0	0	0
CLR	5	8.3	6	10.3	11	9.3
LVX	5	8.3	5	8.6	10	8.5
MTZ	13	21.7	1	1.7	14	11.9
RIF	0	0	6	10.3	6	5.1
TET	0	0	0	0	0	0
double resistance						
CLR + LVX	0	0	3	5.2	3	2.5
CLR + MTZ	4	6.7	0	0	4	3.4
LVX + MTZ	0	0	1	1.7	1	0.8
triple resistance						
CLR + LVX + MTZ	3	5.0	0	0	3	2.5
overall resistance *						
CLR	12	20.0	9	15.5	21	17.8
LVX	8	13.3	9	15.5	17	14.4
MTZ	20	33.3	2	3.4	22	18.6

* combined single, double and triple resistances.

**Table 4:** PCR resistance patterns in Datteln.

	Datteln	
	*n*	%
HP pos. PCRs	111	100.0
no resistance	82	73.9
any resistance	29	26.1
single resistance		
CLR *	13	44.8
FQ	11	37.9
double resistance		
CLR + FQ	5	17.2

* including two cases in which FQ resistance could not be determined.

**Table 5:** Comparison of cultural and genotypic resistance patterns for CLR and LVX/FQ.

Culture		PCR		*n*
CLR	LVX	CLR	FQ	
concordant				
s	s	s	s	42
R	s	R	s	5
s	R	s	R	5
R	R	R	R	3
discordant				
R	s	s	s	1
s	R	s	s	1
s	s	s	R	1

Only combinations with matches: CLR = clarithromycin, LVX = levofloxacin, FQ = fluoroquinolones, s = susceptible, R = resistant.

**Table 6:** EUCAST clinical breakpoints for *H. pylori*.

Antibiotic	Sensitive: ≤ *	Resistant: ≥ *
amoxicillin	0.125	0.250
clarithromycin	0.25	0.5
levofloxacin	1	2
metronidazole	8	16
rifampicin	1	2
tetracycline	1	2

* referring to MIC breakpoints (mg/L); EUCAST clinical breakpoint tables v. 16.0 2026, identical for 2024 and 2025 [36].

## Data Availability

The original data presented in the study are included in the article; further inquiries can be directed to the corresponding author.

## Abbreviations

The following abbreviations are used in this manuscript:

AMS	Antimicrobial stewardship
AMX	Amoxicillin
CLR	Clarithromycin
FQ	Fluoroquinolones (levofloxacin, moxifloxacin)
HP	*Helicobacter pylori*
HUT	*Helicobacter* urease (rapid) test
LVX	Levofloxacin
MTZ	Metronidazole
PCR	Polymerase chain reaction
PPI	Proton pump inhibitor
RIF	Rifabutin/rifampicin
TET	Tetracycline
